# Depressive Symptomatology in Children and Adolescents with Chronic Renal Insufficiency Undergoing Chronic Dialysis

**DOI:** 10.4061/2011/798692

**Published:** 2011-09-20

**Authors:** Edith G. Hernandez, Reyner Loza, Horacio Vargas, Mercedes F. Jara

**Affiliations:** ^1^Pediatric Nephrology Unit, Cayetano Heredia National Hospital, San Martín de Porras, Lima, Lima 31, Peru; ^2^Pediatric Psychiatric Unit, The National Institute of Mental Health “Honorio Delgado-Hideyo Noguchi”, Lima, Lima 31, Peru

## Abstract

This paper presents a descriptive study, using the Birleson Scale to determine the frequency of depressive symptomatology in children and adolescents with chronic renal insufficiency (CRI) undergoing hemodialysis (HD) and chronic peritoneal dialysis (CPD). There were 67 patients (40 female and 27 male) with a mean age of 14.76 ± 2.71 years, duration of illness ≥3 months, 43 (64.18%) patients with CPD and 24 (35.82%) undergoing HD. The frequency of high occurrence, low occurrence, and absence of depressive symptomatology was 10.45% (*n* = 7), 43.28% (*n* = 29), and 46.27% (*n* = 31), respectively; all of the seven (100%) patients with high occurrence of depressive symptomatology were female (*P* = 0.04), and none of these (0%) had a friend to confide in (*P* = 0.03). Depressive symptomatology in patients with CPD was associated with a lower weekly *K*_*t*_/*V* compared to those without depressive symptomatology (2.15 ± 0.68 versus 2.52 ± 0.65; *P* = 0.01). There was no association with patient age, caregiver, time and dialysis type, anemia, bone disease, nutritional or financial status, origin, schooling, or employment.

## 1. Introduction

Depression is a public mental health problem that affects children, adolescents, and adults, negatively impacting their personal, academic, social, and family lives [1]. In recent years the prevalence of depression has increased worldwide, and, at the same time, the presentation age has decreased. According to the World Health Organization (WHO), this makes it the medical condition with the fourth highest loss of life years due to premature death or years living with a severe and chronic incapacity [1].

The definition of depression is a mood alteration characterized by a depressed state of mind, decreased enjoyment and concentration, lack of interest, a feeling of disability, guilt, and hopelessness, insomnia, reduced appetite, and suicide ideation. The earlier it is present, the greater the risk of suicide, substance abuse, and behavioral problems; for this reason it is very important to diagnose and treat quickly [2]. 

The prevalence of depression is about 3–5% in children and around 8% in adolescents, with a female predominance [3, 4]. The presence of depressive symptoms is about 15%. These frequencies increase in patients with a chronic disease [5].

Depression is the most common mental alteration in patients with terminal chronic renal insufficiency, which is neither diagnosed nor studied [5]. Livesley found that the frequency of anxiety, personality disorder, and depression in patients with chronic dialysis was significantly higher than in healthy individuals [6]. Lopes and colleagues found a 20% prevalence of depression in 5256 patients and 13.9% in 9382 patients, both undergoing chronic hemodialysis [7, 8]. Bakr and coworkers compared children undergoing chronic hemodialysis with children not undergoing chronic hemodialysis, finding that 52.6% of the hemodialysis patients had psychiatric alterations, and of these 10.3% had depression. The prevalence of depression changes according to the studied population and the diagnostic method [9, 10].

Different studies show an association between depression, decrease in length of life, and adherence to therapy, with an increase of 15 times the suicide risk and an increasing number of hospitalizations. This makes it an independent risk factor predicting mortality, and one with a significant impact on the patient's quality of life, greater than biological or therapy factors [8, 11]. 

The principal objective of this study was to determine the frequency of levels of depressive symptoms in children with terminal chronic renal insufficiency undergoing chronic hemodialysis and chronic peritoneal dialysis and further to ascertain whether there was a relationship with clinical, demographic, or social factors. We used The Modified Birleson Scale validated in Peru [10]. The adaptation of the original test, Birleson Scale made by Peterson Birleson in England in 1981, used in several studies, was made, and it was evaluated for expert council. It has discharge trustworthiness. 

## 2. Materials and Methods

The investigation was a descriptive, case series study. We evaluated children with terminal chronic renal insufficiency undergoing dialytic treatment for three months, at the dialysis unit of the nephrology service of the Cayetano Heredia Peruvian University.

We selected 84 patients with an average age of 14.76 ± 2.71 years, from which we excluded 14 because they were under 8 years old, two who had Down's syndrome, and one who declined to participate in the study. Sixty-seven patients (40 female and 27 male) fulfilled the inclusion criteria (age between 8 and 18 years, duration of illness ≥3 months, no chronic comorbidities, accepted consent and assent, stable clinically with no psychiatric or psychological comorbidity diagnosed at that time, and knowledge of writing). 43 (64.13%) chronic peritoneal dialysis patients and 24 (35.82%) chronic hemodialysis patients were selected. 

The Birleson Scale was used to determine the presence of depression and depressive symptoms, being the value 13 as the cut-off score, where a score of 13–21 evidenced the presence of depressive symptoms and a value of more than 21 demonstrated depression. While the Birleson Scale has been used in disease states, it still has not been validated in ESRD patients, and therefore high scores are suggestive but not diagnostic of clinical depression. For this reason, those variables were recategorized as high and low occurrence of depressive symptomatology in terms of depression and depressive symptoms, respectively, as well as, an absence of depressive symptoms if the score was <13 and the presence of depressive symptoms ≥13.

Clinical data were collected from the patient charts and sociodemographic data from the patients and their caregivers.

**Statistical Procedure**

The statistical program Stata v.10 was used for the data analysis. Chi^2^ and Student's *t* were employed to determine statistical significance for continuous and categorical variables, and variance analysis was used to compare means.

## 3. Results

**Figure 1:**
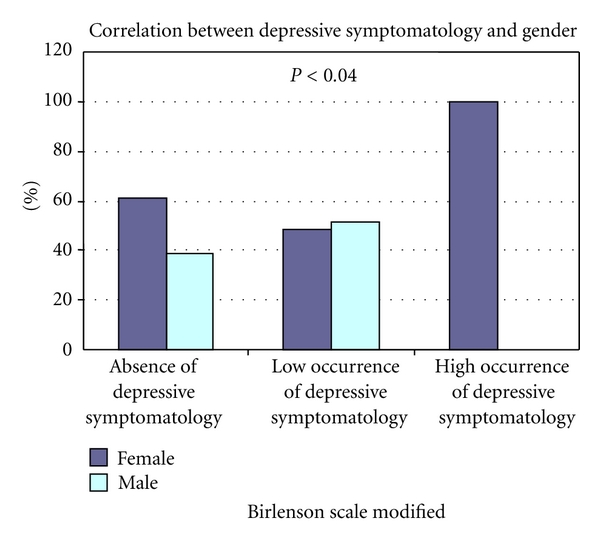
Correlation between depression symptomatology and gender.

The frequency of high depressive symptomatology was 10.45% (*n* = 7), low depressive symptomatology 43.28% (*n* = 29), and no depressive symptoms 46.27% (*n* = 31). Of the children with high occurrence of depressive symptomatology, five (11.63%) were undergoing chronic peritoneal dialysis and two (8.33%) were undergoing hemodialysis. All (100%) of the children with high occurrence of depressive symptomatology were female (*P* = 0.04), and none of the seven (0%) had a friend to confide in (*P* = 0.03) (Table 1 and Figure 1). The occurrence of depressive symptomatology in patients with CPD was associated with a lower weekly *K*_*t*_/*V* compared to those with absence of depressive symptomatology (2.15 ± 0.68 versus 2.52 ± 0.65; *P* = 0.01) (Table 2).

Twenty (83.33%) of the children undergoing hemodialysis were from Lima, and 25 (58.14%) of the children undergoing chronic peritoneal dialysis were from a province outside the capital (*P* = 0.02). Of the patients undergoing chronic peritoneal dialysis, 35 (81.40%) had both parents, but five (11.63%) had only one parent, giving a *P* = 0.027 (Table 3).

We found some relationship between high occurrence of depressive symptomatology and feelings, as follows: not very happy with their physical appearance (4, 57.14%) *P* = 0.04, somewhat with their studies (6, 85.71%) *P* = 0.001, somewhat unhappy with their financial status (5, 71.43%) *P* = 0.001, and somewhat unhappy with their friends (6, 85.71%) *P* = 0.001 (Table 4).

According to the Modified Birleson Scale (Table 5), children with high recurrence of symptomatology answered “always” the following questions: Number 21 (85.7%, *n* = 6) and Number 5, 10, 12, 16, 17, 19, and 20 (100%, *n* = 7). Children with low recurrence of symptomatology answered all questions in a random way, including the questions Number 10 and 21. 

The presence of high and low occurrence of depressive symptomatology was not related to the patient's age, caregiver's age, dialysis period, presence of anemia, bone disease, nutritional status, financial situation, origin, schooling, or employment.

## 4. Discussion

Having a chronic disease during childhood confers a large risk of developing a psychiatric disorder, and chronic renal disease is not an exception: it is a significant stressor with a psychological and social impact on the children and their family [10]. 

In this study, we found a general frequency of 53.73% for high and low depressive symptomatology, 10.45% for high depressive symptomatology and 43.28% for low depressive symptomatology, of whom 11.63% were undergoing peritoneal dialysis and 8.33% hemodialysis.

Bakr et al. [9] analyzed 19 children with chronic renal insufficiency undergoing predialysis and 19 children with terminal chronic renal insufficiency undergoing dialysis, finding a 52.6% prevalence of psychiatric disorders, 18.4% of adaptation disorders, 10.3% of depression, and 7.7% of neurocognitive disorders. However, in children with terminal chronic renal insufficiency, depression was as high as 15.8%; the authors also found a higher prevalence in dialysis patients (68.4%) than in predialysis patients (36.8%). In addition, Fukunishi and Kudo [12] reported that 17 (65.4%) of 25 children with terminal chronic renal insufficiency undergoing peritoneal dialysis had psychiatric disorders; however, they did not analyze the depression frequency. Wass and colleagues [13] found that of 26 British children who were receiving hemodialysis at home, five (19.2%) had a psychiatric disease. Another study, by Garralda and colleagues, showed that psychiatric alterations such as depression are common in children and adolescents (22 children and adolescents with terminal chronic renal insufficiency and 22 predialysis children and adolescents with chronic renal insufficiency, compared to healthy children) and that these increase in those whose renal disease is more severe [14]. 

Children with terminal chronic renal insufficiency have growth retardation and develop secondary sexual characteristics, bone deformities, multiple scars, and so forth. As a consequence of bone dystrophy, uremia, and the treatment, the children look and feel different from other children, thus increasing the risk of psychiatric problems such as depression [15].

Before puberty, depression is more frequent in males, while in the postpuberty period it predominates in females. This is consistent with our results, where all of the children with high occurrence of depressive symptomatology were female and their average age was 14.76 ± 2.71. This differs from other studies that did not find any relationship with gender [4, 10].

Peritoneal dialysis patients without depressive symptomatology had a better *K*_*t*_/*V* value than patients with depressive symptomatology, which was significant (*P* = 0.01). We could infer that depression as comorbidity is a negative clinical factor. Another study did not show any relationship with *K*_*t*_/*V* [16]. There is evidence that suboptimal dialysis increases mortality in patients undergoing peritoneal dialysis and hemodialysis with cardiac, cerebral, and other comorbidities. A *K*_*t*_/*V* that diminishes by 0.1 weekly is associated with a 5% increase in relative death risk [8, 17], and suboptimal dialysis contributes to the risk of depression [11, 18–20]. This finding needs to be clarified in further studies.

None of the children with high occurrence of depressive symptomatology had a friend to confide in (*P* = 0.03), which agrees with other studies. This confirms that social support contributes to the patient's quality of life and decreases the number of hospitalizations in children with chronic renal insufficiency [21].

When asked *How content or happy are you with your physical appearance, studies, financial status, and friends? ( P* < 0.05), they answered “more or less (content or happy)” or “fairly (content or happy).” This confirms the presence of low self-esteem related to high occurrence of depressive symptomatology, as the response “more or less” might be considered “fair” because patients tend to deny depression as a social defense [8, 10].

We know that the stress and impact of disease generate a distressing and downcast reaction and/or pain in children and their families. This is where the depressive symptoms predominate and then develop to depression or other psychiatric changes [22]. It is for this reason that it is recommended that all children with depression, depressive symptoms, and suicidal ideation according to the Modified Birleson Scale or high and low depressive symptomatology according to the recategorization should undergo psychiatric interview to confirm their probable diagnosis. As we know, depression is a clinical diagnosis and the Modified Birleson Scale gives us information about the presence of depressive symptoms, not clinical depression itself. It can be used as a tool to help us to be aware of any possible disorder, which would then need to be confirmed by a psychiatrist. Future research needs to validate self-reported questionnaires in patients with chronic disease like ESRD patients. A few studies show an increased frequency of depression diagnosis after psychiatric interview compared with frequency of possible depression after self-completion of a diagnostic scale [23]. Such individuals should be candidates for treatment, since there is evidence that social, psychological, and pharmacological interventions reduce mortality, morbidity, and treatment withdrawal [24], and improve the quality of life [11, 25] associated with depression in patients with or without comorbidities [26]. Given this, diagnosis of depression should always be included in screening of pediatric patients with terminal chronic renal insufficiency [8].

## 5. Conclusion

The frequencies of high and low occurrence of depressive symptomatology were 10.45% and 43.28%, respectively. The clinical and sociodemographic factors related to high occurrence of depressive symptomatology were female sex, having or not having a friend to confide in, and dialysis dose.

## Figures and Tables

**Table 1:** Correlation of high occurrence of depressive symptoms (score above 21 according to Birleson Scale) and absence of depressive symptomatology (score below 13) with clinical, demographic, and social variables.

Variable	High occurrence of depressive symptoms		Absence of depressive symptoms		*P*
	*n*	%	*n*	%	
*Gender*					
Female	7	100	19	61.29	0.04
Male	0	0	12	38.71	

*Provenance*					
Lima	5	71.43	17	54.84	0.82
Other cities	2	28.57	14	45.16	

*Attending school*					
Yes	1	4.35	14	60.87	0.2
No	6	13.64	17	38.64	

*Relationship age/level of education*					
Yes	4	57.14	13	41.94	0.18
No	3	42.86	18	58.06	

*Are you currently employed?*					
Yes	0	0	4	80	0.49
No	7	11.29	27	43.55	

*Socioeconomic level*					
Extreme poverty	3	9.38	15	46.88	0.4
No extreme poverty	4	17.39	8	34.78	
No poverty	0	0	8	66.67	

*Family*					
Two-parent home	4	8.51	19	40.43	0.28
One-parent home	2	15.38	8	61.54	
Others	1	14.29	4	57.14	

*Anemia*					
Yes	6	11.11	1	7.69	0.16
No	22	40.74	9	69.23	

*Renal osteodystrophy*					
Yes	2	28.57	22	70.97	0.16
No	5	71.43	9	29.03	

*Dialysis type*					
Hemodialysis	2	8.33	13	54.17	0.65
Peritoneal dialysis	5	11.63	18	41.86	

*Peritonitis*					
Yes	1	5	8	40	0.47
No	4	17.39	10	43.48	

*Nutritional status *					
Eutrophic	0	0	4	100	0.4
Mild chronic malnutrition	2	16.67	6	50	
Moderate chronic malnutrition	2	8.7	9	39.13	
Severe chronic malnutrition	3	10.71	12	42.86	

*K_t_/V*					
Acceptable	5	71.43	23	74.19	0.45
Not acceptable	2	28.57	8	25.81	

*Friend to confide in*					
Yes	0	0	17	54.84	0.03
No	7	100	14	45.16	

**Table 2:** Correlation of depressive symptoms (score above 13 according to Birleson Scale) and no depressive symptoms with clinical and demographic variables.

Variable	Depressive symptoms	No depressive symptoms	*P < *0.05
	$\overset{̅}{x}\pm \sigma$	$\overset{̅}{x}\pm \sigma$	
Patient's age	14.67 ± 2.89	14.10 ± 2.78	0.33
Age of patient's guardian	43.38 ± 7.0	40.16 ± 7.44	0.1
Dialysis time	1.91 ± 1.47	2.47 ± 2.41	0.25
Hematocrit (%)	26.52 ± 6.88	28.28 ± 6.44	0.14
Parathormone (pg./mL)	277.37 ± 245.11	309 ± 385.86	0.68
*K_t_/V* hemodialysis	1.45 ± 0.19	1.39 ± 0.28	0.43
*K_t_/V *peritoneal dialysis	2.15 ± 0.68	2.52 ± 0.65	0.01

**Table 3:** Features according to dialysis types.

Variable	Total	HD		DP		*P*
	%	*n*	%	*n*	%	
*Birleson Scale*						
High occurrence of depressive symptomatology	10.45	2	8.33	5	11.63	0.65
Low occurrence of depressive symptomatology	43.28	9	37.5	20	46.51	
Absence of depressive symptomatology	46.27	13	54.17	18	41.86	

*Provenance*						
Lima	56.71	20	83.3	18	41.86	0.002
Other cities	43.28	4	16.7	25	58.14	

*Family*						
Two-parent home	70.14	12	50	35	81.4	0.027
One-parent home	19.40	8	33.3	5	11.63	
Others	10.46	4	16.7	3	6.98	

**Table 4:** Correlation of depressive symptoms with satisfaction with their physical appearance, studies, economic status, and friendship.

Satisfaction with themselves	Very	Somewhat	Not very	*P*
*Physical appearance*				
Absence of depressive symptomatology	48.39%	6.45%	45.16%	0.041
Low occurrence of depressive symptoms	41.38%	10.34%	48.28%	
High occurrence of depressive symptoms	0%	42.86%	57.14%	

*Studies*				
Absence of depressive symptomatology	35.48%	9.68%	54.84%	0.001
Low occurrence of depressive symptoms	17.24%	24.14%	58.62%	
High occurrence of depressive symptoms	0%	85.71%	14.29%	

*Economic status*				
Absence of depressive symptomatology	32.26%	16.13%	51.61%	0.001
Low occurrence of depressive symptoms	0%	34.48%	65.52%	
High occurrence of depressive symptoms	0%	71.43%	28.57%	

*Friendship*				
Absence of depressive symptomatology	58.06%	12.9%	29.03%	0.001
Low occurrence of depressive symptoms	37.93%	13.79%	48.28%	
High occurrence of depressive symptoms	0.00%	85.71%	14.29%	

**Table 5:** Modified Scale of Birleson and frequency of symptoms of the 67 patients; it is presented as an original sample, and it has been literally translated in order to have a better understanding of depressive symptomatology. Always have a score of 2, sometimes of 1, and never of 0.

Questions	Equivalent in English	%		
		Always	Sometimes	Never
(1) Las cosas me gustan, me interesan como antes	I like and am still interested in the same things as before	34.32 (*n* = 23)	59.70 (*n* = 40)	5.97 (*n* = 4)
(2) Duermo muy bien	I sleep well	64.18 (*n* = 43)	31.34 (*n* = 21)	4.47 (*n* = 3)
(3) Me dan ganas de llorar	I feel like crying	11.94 (*n* = 8)	55.22 (*n* = 37)	32.83 (*n* = 22)
(4) Para adolescentes: me gustan salir com amigos. Para niños: me gusta salir a jugar	For adolescents: I like going out with my friends. For children: I like going out to play	38.80 (*n* = 26)	35.82 (*n* = 24)	25.37 (*n* = 17)
(5) Me gustaria escapar, salir corriendo	I would like to run away	10.45 (*n* = 7)	29.85 (*n* = 20)	59.70 (*n* = 40)
(6) Me duele la barriga, cabeza y otros sítios de mi cuerpo	My stomach and my head hurt, as well as other parts of my body	5.97 (*n* = 4)	41.79 (*n* = 28)	52.23 (*n* = 35)
(7) Tengo ganas para hacer las cosas	I have a desire to do things	37.32 (*n* = 25)	50.74 (*n* = 34)	11.94 (*n* = 8)
(8) Disfruto de la comida	I enjoy eating food	55.22 (*n* = 37)	38.80 (*n* = 26)	5.97 (*n* = 4)
(9) Puedo defenderme por mi mismo	I am able to defend myself	43.28 (*n* = 29)	38.80 (*n* = 26)	17.91 (*n* = 12)
(10) Pienso que no vale la pena vivir	I feel that life is not worth living	16.42 (*n* = 11)	28.35 (*n* = 19)	55.22 (*n* = 37)
(11) Soy bueno para las cosas que hago	I am good at the things I do	47.76 (*n* = 32)	41.79 (*n* = 28)	10.44 (*n* = 7)
(12) Me molesto y me irrito por cualquier cosa	I feel bothered and irritated with everything	23.88 (*n* = 16)	55.23 (*n* = 37)	20.89 (*n* = 14)
(13) Disfruto lo que hago tanto como lo hacia antes	I still enjoy doing things as I did before	43.29 (*n* = 29)	47.76 (*n* = 32)	8.95 (*n* = 6)
(14) Me he vuelto olvidadizo y distraído	I have become forgetful and listless	7.46 (*n* = 5)	55.22 (*n* = 37)	37.32 (*n* = 25)
(15) Tengo sueños horribles	I have nightmares	4.47 (*n* = 3)	35.82 (*n* = 24)	59.70 (*n* = 40)
(16) Pienso que haga lo que haga no lograre conseguir lo que deseo o que las cosas no van a cambiar	I think that it does not matter what I do; I will never be able to accomplish the things I want to accomplish; things are never going to change	31.34 (*n* = 21)	31.34 (*n* = 21)	37.32 (*n* = 25)
(17) Me siento muy solo	I feel very lonely	11.94 (*n* = 8)	37.31 (*n* = 25)	50.74 (*n* = 34)
(18) Puedo alegrarme facilmente	I become happy easily	41.79 (*n* = 28)	50.74 (*n* = 34)	7.46 (*n* = 5)
(19) Me siento tan triste que me cuesta trabajo soportarlo	I feel so sad that I can hardly stand it	20.91 (*n* = 14)	28.35 (*n* = 19)	50.74 (*n* = 34)
(20) Me siento muy aburrido	I feel very bored	16.42 (*n* = 11)	58.21 (*n* = 39)	25.37 (*n* = 17)
(21) Pienso muy serio en la muerte o en matarme	I seriously think about death and killing myself	8.95 (*n* = 6)	19.41 (*n* = 13)	71.64 (*n* = 48)

Total				

## References

[1] Atencio BJ, Nucette E, Colina J, Sumaleye S, Gomez F, Hinestroza D (2004). Evaluacion de la depresión y ansiedad en pacientes con insuficiencia renal crónica sometidos a hemodiálisis. *Archivos Venezolanos de Psiquiatría y Neurología*.

[2] Birmaher B, Brent D, Bernet W (1998). Practice parameters for the assessment and treatment of children and adolescents with depressive disorders. *Journal of the American Academy of Child and Adolescent Psychiatry*.

[3] Perera H (2008). Depression in children and adolescents. *The Ceylon Medical Journal*.

[4] Bennett DS, Ambrosini PJ, Kudes D, Metz C, Rabinovich H (2005). Gender differences in adolescent depression: do symptoms differ for boys and girls?. *Journal of Affective Disorders*.

[5] Kimmel P (2002). Depression in patients with chronic renal disease: what we know and what we need to know. *Journal of Psychosomatic Research*.

[6] Livesley WJ (1982). Symptoms of anxiety and depression in patients undergoing chronic haemodialysis. *Journal of Psychosomatic Research*.

[7] Lopes AA, Bragg J, Young E (2002). Depression as a predictor of mortality and hospitalization among hemodialysis patients in the United States and Europe. *Kidney International*.

[8] Lopes AA, Albert J, Young EW (2004). Screening for depression in hemodialysis patients: associations with diagnosis, treatment, and outcomes in the DOPPS. *Kidney International*.

[9] Bakr A, Amr M, Sarhan A (2007). Psychiatric disorders in children with chronic renal failure. *Pediatric Nephrology*.

[10] Vivar R, Pacheco Z, Adrianzen C, Macciotta B, Marchena C (2005). Validation Birleson scale modified for depressive disorders in children and adolescents Peruvian. *Revista Peruana de Pediatría*.

[11] Lew SQ, Piraino B (2005). Psychosocial factors in patients with chronic kidney disease: quality of life and psychological issues in peritoneal dialysis patients. *Seminars in Dialysis*.

[12] Fukunishi L, Kudo H (1995). Psychiatric problems of pediatric end-stage renal failure. *General Hospital Psychiatry*.

[13] Wass VJ, Barratt TM, Howarth RV (1977). Home dialysis in children. *The Lancet*.

[14] Garralda ME, Jameson RA, Reynolds JM, Postlethwaite RJ (1988). Psychiatric adjustment in children with chronic renal failure. *The Journal of Child Psychology and Psychiatry*.

[15] Perrin EC, Gerrity PS (1984). Development of children with a chronic illness. *Pediatric Clinics of North America*.

[16] Amr M, Bakr A, El Gilany AH, Hammad A, El-Refaey A, El-Mougy A (2009). Multi-method assessment of behavior adjustment in children with chronic kidney disease. *Pediatric Nephrology*.

[17] Davies SJ, Bryan J, Phillips L, Russell GI (1996). The predictive value of KT/V and peritoneal solute transport in CAPD patients is dependent on the type of comorbidity present. *Peritoneal Dialysis International*.

[18] Strik JJ, Denollet J, Lousberg R, Honig A (2003). Comparing symptoms of depression and anxiety as predictors of cardiac events and increased health care consumption after myocardial infarction. *Journal of the American College of Cardiology*.

[19] Kimmel PL, Peterson RA, Weihs KL (2000). Multiple measurements of depression predict mortality in a longitudinal study of chronic hemodialysis outpatients. *Kidney International*.

[20] Bloembergen WE, Stannard DC, Port FK (1996). Relationship of dose of hemodialysis and cause-specific mortality. *Kidney International*.

[21] Soliday E, Kool E, Lande M (2001). Family environment, child behavior, and medical indicators in children with kidney disease. *Child Psychiatry and Human Development*.

[22] Kimmel P (2001). Psych osocial factors in dialysis patients. *Kidney International*.

[23] Wuerth D, Finkelstein SH, Ciarcia J, Peterson R, Kliger AS, Finkelstein FO (2001). Identification and treatment of depression in a cohort of patients maintained on chronic peritoneal dialysis. *American Journal of Kidney Diseases*.

[24] McDade-Montez EA, Christensen AJ, Cvengros JA, Lawton WJ (2006). The role of depression symptoms in dialysis withdrawal. *Health Psychology*.

[25] Turk S, Atalay H, Altintepe L (2006). Treatment with antidepressive drugs improved quality of life in chronic hemodialysis patients. *Clinical Nephrology*.

[26] Kimmel PL, Weihs K, Peterson RA (1993). Survival in hemodialysis patients: the role of depression. *Journal of the American Society of Nephrology*.

